# The Road to Developing Standard Time for Efficient Nursing Care: A Time and Motion Analysis

**DOI:** 10.3390/healthcare11152216

**Published:** 2023-08-06

**Authors:** Modi Al-Moteri, Amer A. Alzahrani, Ensherah Saeed Althobiti, Virginia Plummer, Afnan Z. Sahrah, Maha Jabar Alkhaldi, Eishah Fahad Rajab, Amani R. Alsalmi, Merhamah E. Abdullah, Afra Ezeldeen Abduelaal Abduelazeez, Mari-zel M. Caslangen, Mariam G. Ismail, Talal Awadh Alqurashi

**Affiliations:** 1 Nursing Department, College of Applied Medical Sciences, Taif University, P.O. Box 11099, Taif 21944, Saudi Arabia; 2 King Abdulaziz Specialist Hospital, Ministry of Health, Taif 21944, Saudi Arabia; amaalzahrani@moh.gov.sa (A.A.A.); ealthobiti@moh.gov.sa (E.S.A.); efrajab@moh.gov.sa (E.F.R.); maespanola@moh.gov.sa (M.E.A.); taaalqurashi@moh.gov.sa (T.A.A.); 3 Institute of Health and Wellbeing, Federation University, Berwick, VIC 3806, Australia; v.plummer@federation.edu.au

**Keywords:** motion, nurse, observation, time

## Abstract

(1) Background: The amount of time nurses spend with their patients is essential to improving the quality of patient care. Studies have shown that nurses spend a considerable amount of time on a variety of activities––which are often not taken into account while estimating nurse-to-patient care time allocation––that could potentially be eliminated, combined or delegated with greater productivity. The current study aimed to calculate standard time for each activity category by quantifying the amount of time required by nurses to complete an activity category and determine the adjustment time that can be given during work, as well as determine factors that can be altered to improve the efficiency of nursing care on inpatient general wards of a governmental hospital. (2) Method: A time and motion study was conducted over two weeks using 1-to-1 continuous observations of nurses as they performed their duties on inpatient general wards, while observers recorded each single activity, and specifically the time and movements required to complete those activities. (3) Result: There was 5100 min of observations over 10 working days. Nurses spent 69% (330 min) of time during their 8 h morning shift on direct patient care, (19.4%) ward/room activities (18%), documentation (14%), indirect patient care (12%) and professional communication (5%). Around 94 min of activities seem to be wasted and can be potentially detrimental to nurses’ overall productivity and threaten patient care quality. The standard number of hours that represents the best estimate of a general ward nurse regarding the optimal speed at which the staff nurse can provide care related activities was computed and proposed. (4) Conclusions: The findings obtained from time–motion studies can help in developing more efficient and productive nursing work for more optimal care of patients.

## 1. Introduction

Essential to the quality of patient care is the amount of time nurses spend with patients. Literature reports that a higher average of direct nursing care hours allows for more time for patient assessment and lower rates of patient complaints, falls, pressure ulcers and mortality [1,2,3]. Meanwhile, if hospitalized patients received inadequate care time, this may increase their length of stay and the overall use of healthcare resources, while nurse and patient satisfaction decrease, work presenteeism, engagement, patient safety and quality of care also decline [4,5,6,7]. Unfortunately, the literature has shown that nurses spend a considerable amount of time on activities––which are often not taken into account when estimating nurse-to-patient care time allocation––that could potentially be eliminated, combined or delegated to achieve greater productivity [8]. High-quality patient care outcomes cannot be met without efficient nursing staff [9]. Hence, initiatives directed to shift nurse time to patient care are more likely to optimize nurse time and improve patient care outcomes [5].

Nurses’ productivity is also described as nursing efficiency and refers to the quality and quantity of care provided to a patient care group with the same amount and type of resources (e.g., time and staff numbers) [10]. The measure of efficiency of nurses has been established by computing the time required to do each activity incorporating acuity standards [11]. A priority area of healthcare system enhancement plans is improving the productivity of healthcare workers to address the increasing requirements of patients [12], consequently enhancing the quality and safety of patient care. The nurse-to-patient staffing ratio in general wards has been used as an indicator [13], yet shows scant information about how nurses arrange their time to support patient care under that model [14]. If the purpose is to estimate the time nurses spend with patients for care, in order to assess if nurses devote adequate time, then direct methods of assessment are required [9]. Surprisingly little baseline data are available about the proportion of patient care activities time devoted by nurses against the total time allocated to different categories of an average workday [4,9]. This absence of evidence tends to hinder identifying opportunities for improvement.

There is a growing body of evidence which assesses/measures nurses’ work. Of these studies that have tried to estimate the measure of time associated with nurses’ activities in general wards, the activities of patient care have been organized into two major categories: direct and indirect patient care activities [15,16]. Direct nursing activities refer to activities that require direct contact with the patient and/or the patient’s family, meanwhile indirect activities refer to activities related to preparing for direct activities [15,16]. In these studies, direct care nursing was found to take approximately 20–38% of available time [15] and indirect care 11–25% of the time [16]. Other studies have specifically measured the time allocated for unseen activities [17] that have not been concerned with direct or indirect care of a particular patient, but contribute to the management of staff and services such as student and new staff supervision, team communication and reordering unit supplies and equipment [18]. These activities were found to occupy around 3% to 9% of nurse work time [19].

Further, the concept of value-added (VA) was used to calculate the allocated time for nursing activities [16]. Value-adding (VA) refers to any activity that contributes directly or indirectly in a significant way to the patient’s care, such as health assessment and feeding. About 71% of nurses’ work is value-adding care [16]. Other studies measured the time allocated to nursing activities using the nursing scope of practice [20]. In these studies, it was claimed that if nurses were able to implement all activities integral to the nursing scope of practice, then it can be said that the optimal patient care has been met [4]. Nursing activities that were investigated were care planning (18.6%), technical procedures and delegated medical care (5.7%), medication, (6.2%) activities of daily living (6.3%), patient assessment (3.7%), client education (3.3%), communication and coordination (7.5%), miscellaneous/non-healthcare (7.5%) personal time (6.6%), knowledge updating and use (2.5%), relational care (1.2%), staff integration and supervision (1.8%), and optimization of quality and safety of care (0.6%) [4].

Hossny [21] defined four categories of nursing activities that were specific to the nursing workday: (1) patient care activities refer to nursing activities that require either direct (e.g., vital signs measurement, medication administration, cannula insertion) or indirect (e.g., prepare for medication or any procedure, management of referral) contact with the patient and/or the patient’s family; (2) in-ward professional training refers to participation in training and educational activities relevant to the field of patient care such as bedside intravenous (IV) pump training and resuscitation crash cart training; (3) unit refers to all activities relevant to unit requirements such as maintenance of the resuscitation crash cart, cleanliness and orderliness of the nurse station and requisitioning of supplies; (4) personal activities refer to all activities that are relevant to personal needs such as lunch and pray time, or personal telephone calls.

Although many studies have described activity data for nurses working in general hospital settings [22], in most of these studies, the time required to prepare for an activity, the nurse-to-patient ratio and units’ physical layout were not considered. Hence, strategies to solve nursing problems due to ineffective time distribution and management, such as staff shortage and dissatisfaction may be less effective. The current study intends to extend the methodological approaches of data collection and analysis to provide details in the nurse working day and quantify the time spent on each specific activity.

### 1.1. Rationale

This study provides part of a methodological foundation for a larger multi-phase quality improvement research project to ultimately focus on improving the quality of patient care outcomes from the nursing practice perspective, in a governmental hospital in Saudi Arabia. The project included several methods for capturing the quality of patient care outcomes quantitatively and linking nursing practice (i.e., hours of care, behavioral ergonomics, staffing) to patient care quality by: (i) computing standard time for efficient nursing care; (ii) quantifying the biomechanical load and quality of care in nursing; (iii) developing evidenced-based staffing standards using the Workload Indicator of Staffing Needs approach (WISN); and (iv) finally, assessing the effects of the proposed staffing standards on patient care outcomes.

This was mainly achieved by conducting time–motion studies to examine the way an activity was being performed, determine factors that could be altered to reduce unnecessary or excess work, wasteful time and setting up a time standard for carrying out that activity [23]. If a time–motion study helps in reducing the time to perform a certain activity by a certain margin, simply as a result of modification and altering of certain variables without additional expenditure, then efficiency will increase by a corresponding value, equivalent to the reduced time. Indeed, the relationship between efficiency and work studies is clear.

Studies conducted in industries revealed that performance efficiency improved significantly when setting standard time (ST) [24]. ST refers to the “time necessary to perform specific activity by an average skilled worker, working at a typical pace, using a specific method, allowing time for personal needs, fatigue, and unavoidable delay” [25]. In this context, ST may help health institutions develop ergonomic time standards for nurses’ performance [26]. Three values must be considered for ST: “observed time”, “performance rating” and “adjustment time”.

- Observed time is the time recorded by the observer during observation using the appropriate means to calculate the time nurses spend on a specific activity;
- The performance rating factor refers to the nurse’s speed and the efficiency of a nurse in performing a task in comparison to other nurses, and this is expressed as a percentage of the efficiency and may range from 80 to 130% with the standard performance being equal to 100% [26];
- Adjustment time is the additional time added above the basic time to perform the activity and account for delays. It is the most debatable part of calculating ST, because it differs from nurse to nurse, activity to activity, unit to unit, situation to situation, institution to institution and season to season for example. Therefore, specific standardized norms of adjustment time have been set [26]: (i) fatigue adjustment (intended to provide for the physiological and psychological effects of carrying out specified work under specified conditions such as patients with high acuity, traveling time, number of patients, assigned to additional tasks), (ii) personal needs adjustment (intended to provide rest to recover from the physiological and psychological effects, and attend personal needs), and (iii) unavoidable delay adjustment (intended to provide for unavoidable delays such as interruption, multitasking, searching for missing or out-of-stock items, system breakdown, waiting for patients to transfer from the operating room).

**Figure 1:**
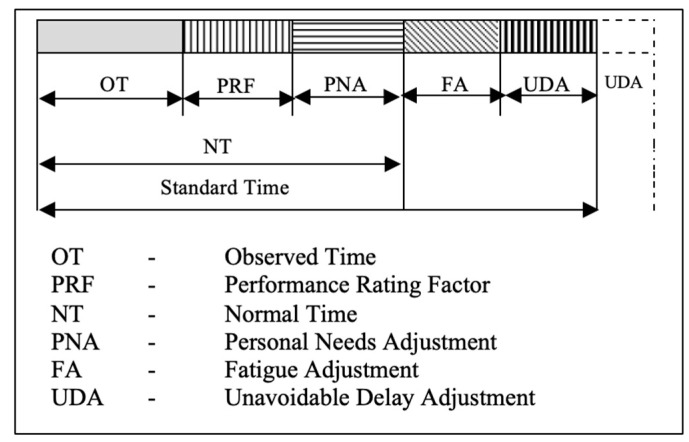
Values of standard time.

The basic ST values are represented by a block diagram in Figure 1.

Establishing a framework and shared language starts by using vigorous metrics and tools to assess staff current work (motions and time) to identify issues affecting the workforce and manage and estimate potential change. This study documents the methodology used to observe a typical 8 h morning shift of a nurse’s work in inpatient general wards of a governmental hospital. The study recorded the amounts of time nurses spent on their different activities using time and motion analysis. The time and motion data collection and analysis technique is generally known to be the gold-standard methodology for accurately measuring a process and developing a workflow that is friendly to the individual body and resources [27].

The technique traditionally involves the shadowing of participants as they perform their duty hours, while observers provide moment-to-moment description of each single activity, along with its exact amount of time spent, using a timekeeping device including electronic stopwatches and videotape cameras, where results are recorded on the observation form [27]. The time and motion data collection and analysis technique is very resource-intensive, as it requires a 1:1 observer-to-subject ratio during data collection. Upon completion of the observations, the observation forms can be compiled and analyzed as near-to-exact representations of participants’ work [27]. This technique has been used both in industry [28] and hospitals [29,30] to provide an objective reliable and valid measure to record the time for completed tasks. This technique permits the development of reliable and valid measures of the exact amounts of time spent on different activities, allowing nursing administrators to understand which activities consume larger portions of nurses’ time [21].

### 1.2. Study Aim

The aim is to calculate standard time for each activity category by quantifying the amount of time required by nurses to complete an activity category and determine the adjustment time that can be given during work, as well as determine factors that can be altered to improve the efficiency of nursing care in inpatient general wards of a governmental hospital.

## 2. Methods

### 2.1. Study Design

This is a time and motion study design, involving 1-to-1 continuous observations of nurses as they perform their duty in inpatient general wards, while observers recorded each single activity along with its duration, more specifically, the time and movements required to complete those activities. Further, notes, comments and other data are recorded. The time and motion observational study is appropriate when researchers intend to record the motion and time required to accomplish a task for the purpose of informing task efficiency [31]. The design involves an external observer who captures detailed data on the duration of a task, using a timekeeping device, for example, an electronic stopwatch or videotape camera [32].

### 2.2. Setting

The hospital in this study is one of the Ministry of Health (MOH) major hospitals and serves the western region of Saudi Arabia. This hospital has 321 beds and provides various specialties, including, but not limited to, internal medicine, surgery, orthopedics, cardiology, oncology and orthopedics. Prior to data collection, a meeting was held with the head nurses of all the general wards—medical, surgical and oncology medical wards—to invite participation of the nurses that met the ward criteria for the current study. An eligible ward was identified by considering all variables that may influence the nurses’ motion and impact the process of calculating the estimated time such as work shift [33], nurse-to-patient ratio [34,35] and ward physical layout [36]. Therefore, the ward criteria were:

- nurse-to-patient ratio should be within the 1:4 range;
- the shift should be an 8 h morning shift, since the nurses’ work peak is evident during the morning shift;
- a long, straight corridor layout.

**Figure 2:**
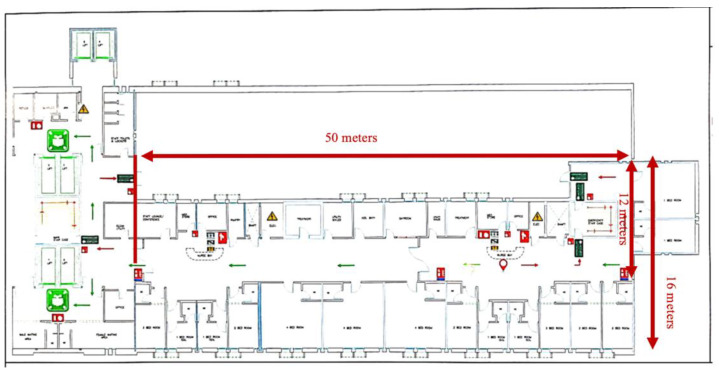
Straight corridor layout.

The ward considered to be the most suitable for the current study is a 29-bed adult medical/surgical oncology ward (MSOW). Patients of the MSOW were mainly admitted through the emergency department (ED) or were transferred from other wards in the hospital. Patients’ acuity of the MSOW range from acute to relatively stable cases and the mean length of stay (LOS) was 7 days (SD: 4.1). Nurses typically work three 8 h shifts per day. The MSOW includes two nursing stations, two offices, one staff lounge conference, one supply room, two medication stores, one pantry, two treatment rooms, two utility soiled rooms and one day room. MSOW includes 15 patient rooms, 4 single rooms, 7 2-bedded rooms and 4 4-bedded rooms. MSOW has a long, straight corridor layout (Figure 2), and the length and width of the ward is 50 m and 12–16 m, respectively. To control the time required for nurses to move between their patients, the time duration required to travel along the 50 m length corridor was estimated to be: 50 m/0.81 = 62 s. All licensed nurses working at MSOW at the time of the study (N = 29) were considered potentially eligible to participate, and they were invited if they met the inclusion criteria. Th MSOW patient ratios at the study time typically ranged from 1:3 to 1:6, depending on patient acuity.

### 2.3. Recruitment

Upon receiving all permissions and approvals from the Hospital Review Board and MSOW head department, and prior to conducting the study, all nurses working on the MSOW at the time of the study were approached and invited (N = 29). First, an invitation letter was attached on the MSOW’s bulletin board inviting all nurses working in the MSOW to join an informative online session in a Zoom Room using a QR code if they were interested. Second, the online session was held to introduce the research project and explain the eligibility criteria. Researchers invited potentially eligible nurses who were: (i) registered nurses (RNs), (ii) scheduled on the morning shift, (iii) providing direct nursing care for patients, and (iv) work experience in the MSOW equal and not less than six months. Out of the 19 nurses who attended the online session, 13 met the inclusion criteria and 10 RNs agreed to participate. Written informed consent was obtained from participants prior to being observed.

### 2.4. Observational Tool

In preparation to define the set of nursing-specific activities to assist in the current study, a pilot study was conducted using a direct observation approach. During the pilot study, all activities that encompass a nurse’s work during a morning shift were noted, and additional notes were written if needed. Around 56 activities were defined and assigned to one of six general categories. The six general activity categories are consistent with other literature [21] in the field to allow for cross-comparisons to be made. The description of each category and related activities are specified below:

- Direct patient care, which involves activities such as health assessment, vital signs, medication, patient comfort, procedures;
- Indirect patient care, which involves activities such as handover, doctor rounds, diagnostic tests;
- Documentation, which involves activities such as paper-based charting, electronic-based charting;
- Professional communication, which involves activities such as exchange information, in-hospital phoning, refereeing;
- Unit/room, which involves activities such as bed-making, room- and nursing station-tidying, infection control measures, supplies maintenance, unit learning;
- Personal, which involves activities such as self-learning, praying, eating, visiting administrative offices, personal phone calls, aside-chatting.

For the observation tool, one of the research authors first accessed the Web-based time and motion validated data collection tool [37], TimeCaT (accessed on 1 May 2023 https://app.timecat.org/), by creating an account, and then invited other observers as data collectors. The TimeCaT data collection instrument is a three-dimensional unique approach that allows for the capture of the activity from multiple dimensions (communication, task and location) and measures of multiple concurrent activities, adding comments and exporting study reports.

However, in the current study, the dimension of communication and location were not used. Instead, communication was classified as an activity in the patient-related care task dimension. The three dimensions were re-defined to represent: (i) time-consuming/wasteful activities (which involve activities related to interruption and traveling), (ii) patient-related care tasks (which involve all activities listed under direct and indirect patient care, documentation, professional communication), and (iii) non-patient-related care tasks (which involve all activities listed under unit/room, personal needs, miscellaneous). Eventually, the list of the defined 56 nursing activities was entered in the three dimensions of TimeCaT. Further, TimeCaT allows for observers to enter notes attached to each activity. Hence, observers used this feature to record activity location, who the nurse communicated with when communication was selected as the activity and record types when documentation was selected as the activity.

### 2.5. Observer and Inter-Observer Reliability Assessment

Prior to the data collection process, 14 observers—8 holding a BSN degree and 6 nurse interns—attended trial observations for at least one shift (8 h). The study purpose, data collection procedure and protocol, the TimeCaT data collection tool, and definition of each activity and category were presented. Because of observers’ clinical experience, they were able to easily identify and differentiate various nursing activities. TimeCaT interface involves a feature to calculate the kappa coefficient for inter-observer reliability. Hence, observers had two rounds of inter-observer reliability to assess whether observers provided consistent estimates of the type and the time of activities. Cohen’s kappa coefficient was 0.72, indicating strong inter-observer agreement.

### 2.6. Data Collection Procedure

Table 1 presents the study protocol. A typical 8 h morning shift was divided into four divisions: 07:00 a.m.–09:00 a.m., 09:00 a.m.–11:00 a.m., 11:00 a.m.–01:00 p.m. and 01:00 p.m.–03:30 p.m. Observation time divisions aimed to prevent observer fatigue. Observation schedule was developed prior for all nurses who agreed to participate according to the ward staffing schedule. Over a period of two weeks, 10 nurses for 10 morning shifts were directly and continuously observed by 14 trained observers using the nursing-specific activity list that was created in the TimeCaT three dimensions. MSOW nurses were reassured that the observation would bear nothing on their evaluation or quality insurance reports. The time and motion study records were exported as an excel sheet and saved. Every day, one nurse was to be shadowed for the entire shift. To maintain continuity of the observations, it was not allowed for one observer to disconnect observation unless the second observer arrived and assumed the observation. To collect data reflecting real time, no interaction between observers and the observed registered nurse, nor interruption during the observation were allowed. Further, observers kept a distance from the observed registered nurse.

### 2.7. Analysis

Ten Excel spreadsheets were exported (one per each observed day) from TimeCaT for statistical analyses. The sheets were reviewed and organized manually according to the six categories considering the qualitative comments and notices. Then, researchers compiled these sheets and counted the grand total sheets (one grand sheet for all days). After these steps, a descriptive analysis was conducted, and the percentage of time spent on activity and category were calculated. In addition, the number of interruptions and duration of traveling were determined.

## 3. Results

### 3.1. Time Allocated

All participants were female RNs and their educational background was a BSN degree, with a minimum of six months of work experience in the MSOW. The nurse-to-patient ratio varied from a minimum of 1:3 to a maximum of 1:6 based on patient acuity. The time and motion data collection process over a two-week period covered a total of 5100 min. Table 2 shows the relevant portion of the activity category results of the nurse’s duty during the 8 h shift. Table 2 shows that activities related to direct patient care category consumed more time during a shift than the indirect patient care category. The direct patient care category includes activities that require direct contact and interaction with the patient, such as measuring vital signs, performing health assessments and cannulation. Conversely, the indirect patient care category did not require interaction or direct contact with the patient and might be performed outside the patient room. In addition, there was a notable percentage of time that a nurse completed (i) ward/ room-related activities and (ii) charting and documenting. Non-nursing, patient and unit activities (miscellaneous activities) are examples of activities that may not take much time during a shift.

Table 3 included personal need and affair activities, eating, drinking, praying, non- patient-related computer use and other activities. During the 8 h morning shift, nurses spent 12% of the total time, with an average of 58 min per morning shift.

Although the portion of time associated with traveling between locations on the ward may not be as large as the six nursing activity categories, Table 4 presents that moving between two patient rooms consumes a significant amount of time.

### 3.2. Observational Comments

Observers noted that there were frequent interruptions (60 times) that accounted for a total of delay 73 min per an 8 h morning shift (Table 5). All activities related to the direct patient care category were interrupted. Within this category medication was the most interrupted activity (16 times per total time allotted for medication) and accounted for a delay of around 00:14:43. Although activities related to patient comfort were less interrupted compared to medication (7 times). The interruption caused a similar amount of delay of 00:14:18. Further, almost all activities related to indirect patient care category were interrupted. Within this category, handover tasks were the most interrupted (6 times), with a delay accounting for about 00:06:46. The remaining interruption varied from a few seconds to a few minutes. Additionally, observers noted that nurses mainly multitasked by carrying out more than one task at the same time. Indeed, there were plenty of cases for multitasking noted by observers, including, but not limited to, preparation for a procedure while completing some paper-based forms at the same time, assessing pain while changing IV fluid, communicating with patient relatives while preparing medication, searching patient file as per doctor request while administering IV fluid, explaining discharge instructions while checking vital signs, etc. In this study, although observers used TimeCaT, they encountered difficulties to separately calculate the time between when one activity ended and another began. Hence, the calculation was not applied to the new task that interfered with one already started. Multitasking was noticed mainly among most activities related to direct and indirect patient care and documentation categories. Specifically, professional communication between nurses and patients, family, nurses, workers and other health professionals, while administering medication, assessing patient, and documenting were common multitasks.

Observers also noted that the observed nurses mainly did some additional tasks that were unrelated to nursing, the patient or the unit. These miscellaneous activities accounted for 20 min per the 8 h morning shift (Table 2). Observers also noted that the observed nurses mostly cared for patients in rooms scattered across the ward. Hence nurses travel long distances between their patient rooms, which may add additional delay. The above-mentioned delays have been reflected in the time nurse left the work. Indeed, observers noted that some of the observed nurses remained at work up to 16:30 p.m. to complete unfinished activities and the most noted left uncompleted activity was documentation. Summing the total time presented in Table 2, Table 3, Table 4 and Table 5 revealed that during a typical 8 h shift, a nurse spends 550 min at work, which exceeds the 480 min.

### 3.3. Calculating the Standard Time (Effective Minutes)

Little differentiation in terms of nursing activities and workflow is presented among all general units and wards in the studied hospital, and therefore standardized nursing activity time can be constructed and generalized.

Step 1—To determine the observed time, the total observed time of each activity category that occurs consistently throughout the 10 days (during the morning shift) was compiled and divided by the 10 to identify the average time per an 8 h shift. The average observed time for nursing activities without interruption = 330 min.

Step 2—In the current study, for nurses to work at 69% (330 min) performance rate and at the same time be efficient, they would be required to work more than eight hours. Meanwhile, for nurses to work at 105% performance rate, they would be required to work for 7.6 h (456 min) (Equation (1)).

**Equation (1)** Effective hours

(1) $$\begin{array}{cccc} 8\;h & \; & 8\;h & \\ ----------- & =12\;h & ----------- & =7.6\;h\\ 69\% & \; & 105\% & \end{array}$$

In general, to calculate how many hours would be required if nurses work at the rate of <100% (less efficient), 100% (standard), >100% (efficient) is something of an art [25]. In this study, to calculate the performance rating factor, the observed time of a selected activity (without interruption) was divided by the normal performance reported in the literature of that activity. In the current study, vital signs observed time was selected as a standardized activity and compared with the time reported in the literature [22], as well as confirmed with the department head and charge nurse (Equation (2)). Hence, the performance rating factor was 105%.

**Equation (2)** Performance rating factor

(2) $$\begin{array}{cc} 00:05:22 & \\ ----------- & =1.052288\\ 00:05:10 & \end{array}$$

Step 3—To compute the normal time of the total activity categories, the total observed time was multiplied with the performance rating factor using the following formula: “Normal time = (average observed time) (performance rating factor)”.

Step 4—The adjustment percentage was estimated by calculating the time spent on personal needs, interruptions, traveling and miscellaneous activities. As mentioned above, standard time includes some additional time, called the adjustment time: (i) personal needs such as rest, eating, drinking, etc; (ii) fatigue, due to physical movement (traveling), acuity of patient and mental stress, and (iii) unavoidable delays due to interruption by others, or delays due to searching for missing or out-of-stock items, assigned to new admission, etc. Although in the current study personal needs, fatigue and unavoidable delay represented 12%, 14 and 19%, respectively, our calculation demanded only 7% for personal needs, 5% for fatigue delay, making a total of 12% (around 1 h) [16]. However, unavoidable delay adjustment is known to be set based on the actual study results [22,38,39]. Around 93 min (20%) (interruption and miscellaneous activities) were reported as a delay and ranged across the different activity categories. Consequently, the unavoidable delays were adjusted based on the frequency and lengths of the interruption from 5 to 20%**.**

Step 5—To calculate the standard time nurses spend to complete each activity category after considering the adjustment time, researchers used the following formula: Standard time = normal time/1-adjustment factor. The ST for each nursing activity category will be as shown in Table 6.

## 4. Discussion

This study aimed to calculate the standard time (effective minutes) for nurses to be more efficient in undertaking their tasks. Establishing standard time (ST) can indeed be used as the basis for improvement plans. A time–motion study is thought as a first step on the path to change. Indeed, the study design per se allows researchers to test distinct interventions targeting nurses’ activities in the studied hospital in order to modify their time allocation and then compare post-interventional changes in the second phase.

In general, the current study findings show that nurses spent 69% of their time on patient care-related activities concerning direct care (19%), indirect patient care activities (12%), documentation (14%) and unit (18%) and professional communication (5%). The results are in accordance with previously published studies [21]. Indeed, the percentage was almost similar to that found by Upenieks et al. [40], but slightly less than in a study by Antinaho et al. [16]. The much-larger proportions of time were devoted to both documentation and unit/room-related activities, making a total of 36% compared to 31% devoted to direct and indirect patient care activities. Perhaps having larger proportions of time dedicated to documentation in the current study is due to the use of paper-based routine documentation.

Numerous motion studies have integrated communication and documentation within indirect patient care activities category [15,16], while others classified them as a separate activity category [21,41]. Categorizations in the current study were developed based on a time and motion study of nurses by Hossny [21]. In general, in the current study, the decision to integrate or separate communication and documentation activities has been based on the estimated weight of these two activities in the studied context during the phase of preparation to define the set of nursing-specific activities.

The efficiency of nursing activities is a multi-factorial problem, and solutions remain varied and controversial. It can be argued that computing standard time for nurses’ work for the purpose of determining the required staffing is not that simple a task due to the nurses’ workload, variations in nursing care demands and complexity of the nursing work environment. However, the World Health Organization [42] has encouraged shifting the direction toward the inclusion of standard time and adjustment time among others in estimating staff requirements. Since then, several countries, e.g., Iraq [43], Oman [44], Namibia [45] and India [46], have attempted to use such an approach. In contrast to the current study, most works in the above-mentioned countries did not use a real-time observation approach; instead, they used census and annual statistics and reports that were regularly collected to estimate the time. Hence, direct comparison between the current study and previous works is intriguing due to differences in approaches and methodologies.

While it is assumed that all nurses perform the same activities and work under the same working conditions, some nurses may work more efficiently than others. As such, it is difficult to judge whose work should be considered efficient if standardized time is not known [4]. In this study, it seems that the nurses were less efficient as they were unable to accomplish their work activities with less waste. The goal is to be more efficient in the same allotted time. The current study results show that various nursing and work practices can be subjected to the process of change and improvement.

In the current study, it was typical for nurses to be assigned patients with greater distances between their rooms. This has mandated nurses to spend a significant amount of their time—69 min—walking during the shift, which adds to their wasted time. “Geographical patient-bed assignment” refers to the “location of beds for patients assigned to a nurse for the duration of a shift” ([47], p. 24). Although the concept of “Geographical patient-bed assignment” has been investigated profoundly to address nurses’ fatigue, dissatisfaction, burnout and workload problems [48,49,50], it is often overlooked in regards to its impact on nurses’ productivity [47]. Although deciding upon patient-bed assignments for each nurse in the ward can sometime be challenging [51], Acar and Butt [49] asserted that the head nurse who assigns nurses to patients in the ward must consider the patient bed location in addition to the level of patient acuity. Given the fact that most hospitals now often operate at a full or nearly full patient bed occupancy, there is a need to quantify the impact of considering geographical patient bed assignment on nurse productivity.

Second, of the recorded time, 20% (93 min) was considered as delayed time and are clearly targets for improving efficiency. Physicians during their visit to the ward disorganize patient files and keep it opened at nursing station(s). Time spent arranging patient files after they have been disorganized by other health professionals is equivalent to two tasks of direct patient care activities (e.g., assessing patients and measuring vital signs). If physicians returned the patient files to their correct location, nurses would possibly have an 11 min delay period advantage. The literature has shown that collaboration between nurses and physicians is fundamental to have positive treatment outcomes, thus improving the quality of healthcare [52].

Third, documentation represented a significant proportion of nursing time (14%), but remained in line with the literature [16]. The studied ward uses inpatient stay paper-based records; perhaps it may be useful to invest in healthcare information technology systems (ITS). Previous studies show the significant impact of using ITS in saving nurses’ time and, therefore, increasing nurse work efficiency [53]. Indeed, researchers reported that the time saved on documentation due to ITS installation was spent on direct patient care tasks [53].

Fourth, using the principles and design methods of a human-factors approach, which focuses on how to make the best use of human capabilities by modifying or simplifying job tasks and activities to optimize nurses’ overall performance [54], the following queries can be proposed: (1) can the activity be eliminated? (e.g., arranging patient files, traveling to different wards); (2) can the activity be combined and completed alongside another activity? (e.g., physical assessment performed whenever there is direct contact with the patient for a different activity); (3) can the activity be simplified and completed in an easier way? (e.g., using charting by exception, investing in ITS).

It can be said that this study helps in some way to form clear descriptions of nurses’ activities, explore how they spend their time, set standard times and determine adjustment times. Such information indeed would help to initiate in-depth reflections on nursing care and identify areas for improvement [16].

Although the current study is concerned with waste elimination and productivity improvement, some results are noteworthy for discussion, such as interruptions and multitasking, which can break the continuity of the activity, causing temporary pause and significant delays. While interruptions sometimes may be useful to maintain patient safety outcomes [55], most are not. [4]. This study revealed that the observed nurses were most likely to be interrupted and/ multitasked during direct patient care, specifically during medication administration. The study findings are aligned with the literature [9,56] and raise patient safety issues. Accurate capture of unnecessary interruptions and identification of how these can be eliminated are recommended.

### Limitations

There are some limitations to this study. Observation occurred only during morning shifts. There is a possibility that the study results may not represent the nursing work taking place during the evening and night shifts, or over weekends. This study was carried out in one large regional hospital at Saudi Arabia, and represented one inpatient unit for two clinical wards (oncology medicine and oncology surgery). Hence, generalization of the study findings should be taken with caution within other populations. The strengths of the study include using a time–motion study design.

## 5. Conclusions

Using the time–motion design to comprehensively measure the time allocated by nurses to different work activities has allowed for authentic information to be collected. The study findings reveal that in the situation, for nurses in the studied ward to work efficiently, they would be required to work more than an 8 h shift. These findings suggest the need to improve the efficiency of nursing work to support nurses in the direct care of patients, by (1) reviewing inpatient stay paper-based records and/or installing ITS, (2) carefully planning the patient–nurse assignment, (3) using ergonomic principles and (4) enhancing the nurse–physician collaborative relationship. The next step should be to test these proposed solutions to create a more effective work environment. Future research is recommended to include interviews with nurses so that the richness of their views can be heard.

## Figures and Tables

**Table 1:** Study protocol.

Criteria	Study Protocol			
Purpose	Baseline data for nursing activities	How nurses spent their break time	Nurse location and movement	Miscellaneous work
Data collected	All patient and unit activities during the 8 h morning shift	All personal activities during the 8 h morning shift	Travel time during the 8 h morning shift	All non-patient and unit activities during the 8 h morning shift
Values	Observed time	Time for personal needs	Fatigue time	Unavoidable delay time
Study period	All on-shift hours for ten days	All on-shift hours for ten days	All on-shift hours for ten days	All on-shift hours for ten days
Collecting tools	Digital assistants Excel sheet	Digital assistants Excel sheet	Digital assistants Excel sheet	Digital assistants Excel sheet
Observation division	2 h observation time division	2 h observation time division	2 h observation time division	2 h observation time division
Method	For each nursing activity Track nurse Select category Note activity duration Note if there is interruption (frequency)	For each observed nurse o Track nurse o Note rest duration	For each observed nurse o Track nurse location o Note travel duration	For each non-nursing patient and unit activity o Note the duration
Participation	All nurses agreed to participate	All nurses agreed to participate	All nurses agreed to participate	All nurses agreed to participate
Nurse shift studied	Morning shift	Morning shift	Morning shift	Morning shift

**Table 2:** Percentage of nurse time spent in activity category during the 8 h shift.

Activity Category	Multitasking and/or Interruption	Time and Percentage of the 8 h Shift Spent on Activities in Category
Direct Patient Care Category		(93 min) 19.4%
Health assessment (charting not included)	Noted	00:07:27
Vital sign measurement and preparation (charting not included)	Noted	00:11:21
Medication including preparation and administering (charting not included)	Noted	00:36:26
Patient comfort care (e.g., daily living activities, feeding, answer Qs, fixing tubes)	Noted	00:32:31
Procedures including preparation time	Noted	00:06:28
Cannulation and intravenous fluids, including preparation, insertion and hanging	Noted	00:05:21
Indirect Patient Care Category		(58 min) 12%
Handover (endorsement) including morning, admission, during and end of the shift	Noted	00:33:20
Doctor rounds	Noted	00:19:13
Diagnostic test including collecting and sending	Noted	00:04:09
Others		00:00:55
Documentation		(68 min) 14.2%
There were 15 distinctive inpatient stay paper-based records	Noted	
**Professional Communication**		**(24 min) 5%**
W. Staff nurse (s)		00:06:58
W. Doctor (Dr rounds not included here)		00:03:07
W. Head nurse		00:04:09
W. Charge Nurse		00:00:54
W. Nurse Supervisor		00:00:35
W. Clinical Instructor		00:00:55
W. patient’s relative/watcher (including educating, explaining and instructing)		00:01:58
W. Patient Care technician (PCT)		00:01:09
Answer in-hospital call		00:01:54
Make in-hospital call		00:02:41
Ward/room mainly based on assignment		(87 min) 18%
Apply infection control measures including, hand washing, rubbing, masking, donning and doffing personal protective equipment (PPE)		00:03:01
Checking point of care testing (POCT) [assigned task]		00:13:27
Arrange nurse stations [nurse who has free time and willing to do]		00:08:29
Check and arrange medical equipment room [assigned task]		00:17:56
Arrange the medication supply room [nurse who has free time and willing to do]		00:09:21
Arrange admission and discharge logbook census		00:02:01
Check patient room safety including curtains and electrical wiring [assigned task]		00:02:58
Sending empty ampules of narcotic to the pharmacy [nurse who has free time and willing to do]		00:02:00
Checking narcotic checklist [nurse who has free time and willing to do]		00:04:08
Check the supply with the head nurse [nurse who has free time and willing to do]		00:12:33
Others		00:01:36
Total		330 min out of the 480 min (69%)
Miscellaneous activities		00:20:41 (4.3%)
Searching for missing or out-of-stock item		00:07:47
Printing forms		00:01:43
Arrange patient files after doctors’ rounds and visits		00:11:11
Total time in minutes (%)		350 min

**Table 3:** Percentage of time spent by nurses in activities that related to their personal needs.

Personal Needs	Time and Percentage of Nurse Travel Path
Rest, coffee, eat, drink and pray	00:42:24
Personal affairs, emailing/texting, calling/helping friend(s)	00:05:55
Non-patient-related computer use	00:04:58
Chatting	00:06:07
Totals	(58 min) 12%

**Table 4:** Most frequently traveled paths by nurses during an 8 h shift.

Travel Path	Time and Percentage of Nurse Travel Path
Moving between the two nursing stations	00:06:16
Moving between patient room and nursing stations	00:04:08
Moving between two patient rooms	00:36:30
Moving between patient room and supply/utility room	00:06:11
In-transit (traveling to pharmacy, X-ray and so on)	00:16:59
Totals	(69 min) 14%

**Table 5:** Interruption duration.

Activity Category	Freq. of Interruption	Duration
Direct patient care category	33 times	00:43:28
Indirect patient care category	10 times	00:10:06
Documentation	17 times	00:19:38
Total		73 min (15%)

**Table 6:** The standard time.

Activity Category		Observed Time	Calculation ST	Unavoidable Delay Adjustment	ST (Normal Time and Adjustment Time)
1	Direct patient care activities	93 min	(93) (1.05) = 97.65 min 97.65/1−0.20 = 122 min.	20%	122 min
2	Indirect patient care activities	58 min	(58) (1.05) = 60.9 min 60.9/1−0.10 = 67.6 min.	10%	68 min
3	Documentation	68 min	(68) (1.05) = 71.4 min 71.4/1−0.15 = 84 min.	15%	84 min
4	Professional communication	24 min	(24) (1.05) = 25.2 min 25.2/1−0.05 = 26.5min.	5%	27 min
5	Ward/room	87 min	(87) (1.05) = 93.4 min 93.4/1−0.05 = 98.3 min.	5%	98 min
6	Miscellaneous activities	20 min	Imbedded with the activities		
7	Personal needs adjustment	58 min	7%	-	33 min
8	Fatigue adjustment	69 min	5%	-	24 min
9	Unavoidable delay	73 min	Imbedded with the activities		
	Total	550 min			456 min 7.6 h

## Data Availability

All data generated or analyzed during this study are included in this published article.
